# Adherence to the neonatal resuscitation algorithm for preterm infants in a tertiary hospital in Spain

**DOI:** 10.1186/s12887-018-1288-3

**Published:** 2018-10-09

**Authors:** Silvia Maya-Enero, Francesc Botet-Mussons, Josep Figueras-Aloy, Montserrat Izquierdo-Renau, Marta Thió, Martin Iriondo-Sanz

**Affiliations:** 1 Neonatology Service, Hospital Clínic, seu Maternitat, ICGON (Institut Clínic de Ginecologia, Obstetrícia i Neonatologia), Barcelona University, Sabino de Arana, 1, 08028 Barcelona, Spain; 2 Neonatology Service, Hospital Sant Joan de Déu, BCNatal (Centre de Medicina Maternofetal i Neonatal de Barcelona, Hospital Sant Joan de Déu, Hospital Clínic), Barcelona University, Passeig de Sant Joan de Déu, 2, 08950 Esplugues de Llobregat, Barcelona, Spain

**Keywords:** Neonatal resuscitation, Video recording, Very preterm infant, Delivery room

## Abstract

**Background:**

There is evidence that delivery room resuscitation of very preterm infants often deviates from internationally recommended guidelines. There were no published data in Spain regarding the quality of neonatal resuscitation. Therefore, we decided to evaluate resuscitation team adherence to neonatal resuscitation guidelines after birth in very preterm infants.

**Methods:**

We conducted an observational study. We video recorded resuscitations of preterm infants < 32 weeks’ gestational age and evaluated every step during resuscitation according to a score-sheet specifically designed for this purpose, following Carbine’s method, where higher scores indicated that more intense resuscitation maneuvers were required. We divided the score achieved by the total possible points per patient to obtain the percentage of adherence to the algorithm. We also compared resuscitations performed by staff neonatologists to those performed by pediatricians on-call. We compared percentages of adherence to the algorithm with the Chi-square test for large groups and Fisher’s exact test for smaller groups. We compared assigned Apgar scores with those given after analyzing the recordings and described them by their median and interquartile range. We measured the interrater agreement between Apgar scores with Cohen’s kappa coefficient. Linear and logarithmic regressions were drawn to characterize the pattern of algorithm adherence. Statistical analysis was performed using SPSS V.20. A *p*-value < 0.05 was considered significant. Our Hospital Ethics Committee approved this project, and we obtained parental written consent beforehand.

**Results:**

Sixteen percent of our resuscitations followed the algorithm. The number of mistakes per resuscitation was low. Global adherence to the algorithm was 80.9%. Ventilation and surfactant administration were performed best, whereas preparation and initial steps were done with worse adherence to the algorithm. Intubation required, on average, 2.2 attempts; success on the first attempt happened in 33.3% of cases. Only 12.5% of intubations were achieved within the allotted 30 s. Many errors were attributable to timing. Resuscitations led by pediatricians on-call were performed as correctly as those by staff neonatologists.

**Conclusions:**

Resuscitation often deviates from the internationally recognized algorithm. Perfectly performed resuscitations are infrequent, although global adherence to the algorithm is high. Neonatologists and pediatricians need intubation training.

## Background

Neonatal resuscitation (NR) is the most frequently performed resuscitation in hospitals [1–3]. Infants that are more immature are more likely to require support. Approximately 85% of very preterm neonates need intervention during transition after birth and their viability and prognosis greatly depend on the care they receive in the delivery room (DR) [4–6]. Most preterm infants initiate breathing after birth, but they often have a weak, insufficient respiratory drive. Guidelines recommend tactile stimulation (warming, drying and rubbing the back or soles of the feet) to stimulate breathing. Guidelines exist to standardize and optimize resuscitation. However, there is evidence that the sequence and quality of interventions during NR often deviate from guidelines [3, 7–11]. Video recording has been widely used for educational and clinical quality assessment purposes, with good acceptance by caregivers [12, 13]. It is inexpensive, it does not interfere with resuscitation, and it offers data to assess performance accurately. Video reviewing reinforces teamwork and permits identification and amendment of errors that otherwise could be neglected. Combining the recording of physiological parameters (ECG, pulse oximetry (PO), capnography and respiratory function monitoring) with video images helps audit performance [12–15]. There is a lack of information about adherence to NR guidelines in Spain. Consequently, we sought to evaluate adherence to NR guidelines in very preterm neonates at our hospital. Our main hypothesis was that resuscitation often deviates from the algorithm. A secondary hypothesis was that staff neonatologists perform better than pediatricians on-call because they work only with neonates and have more experience on average, whereas pediatricians are younger and work with children up to 18 years.

## Methods

We conducted this observational study at Hospital Clínic de Barcelona, a tertiary referral center in Spain where approximately 150 babies < 32 weeks’ gestational age (GA) are born every year. Our Hospital Ethics Committee approved this project. We recorded and analyzed these infants’ resuscitations after obtaining written parental informed consent. We aimed to analyze as many NRs as possible. However, given the difficulties in obtaining parental consent in such moments of stress, we aimed to analyze a representative sample of at least one-third of all NRs performed. Thus, we decided to record 50 resuscitations. We had planned to obtain the data in 1 year, although it took us longer (16 months), as fewer candidates were born during the study period than expected. This study was the basis for the doctoral thesis of the main author (see link in http://www.ub.edu/medicina/doctorat/lectura.htm May 27, 2011). However, the data were never published. The authors believe that the results and conclusions may be perfectly applicable today.

Inclusion criteria: All babies < 32 weeks GA were candidates for inclusion in this study. When the pediatrician was required in the delivery room, parents were approached for consent to record the NR. After obtaining written consent, the resuscitator began recording the NR, and that case was included in the study.

All infants were resuscitated under a radiant heater equipped with a neonatal automatic ventilator (Babylog 2, Dräger Medical, Drägerwerk AG & Co. KGaA, Lübeck, Germany) that included an oxygen blender and could provide Continuous Positive Airway Pressure (CPAP) and Positive Pressure Ventilation (PPV) and with a pulse-oximeter (Nellcor™ NPB-295, Minneapolis, MN, USA). A Sony Handycam DCR-SR 32 E (Sony, Tokyo, Japan) digital video camera attached to the upper left side of the radiant warmer recorded the newborn, the hands of the resuscitators and the PO screen. The clinical team turned the recording on before the baby was born.

**Fig. 1:**
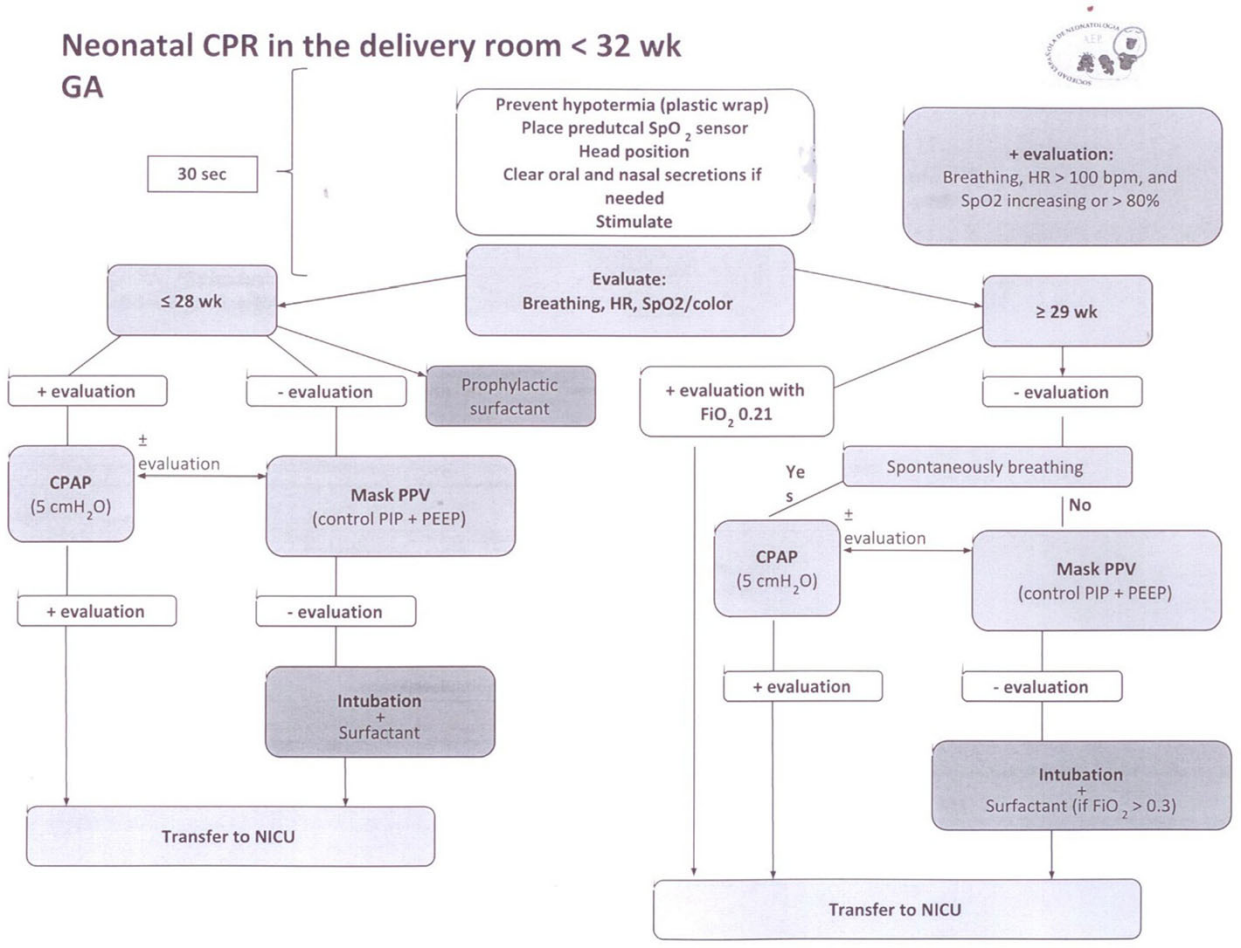
Algorithm of the Spanish Society of Neonatology for the resuscitation of the very preterm infant. Spanish Society of Neonatology, 2007. Obtained from http://www.se-neonatal.es/Comisionesygruposdetrabajos/GrupodeRCPNeonatal/tabid/76/Default.aspx#Publicaciones

We designed an evaluation sheet to score 12 domains in each resuscitation (Table 1) according to the algorithm of the Spanish Society of Neonatology, adapted from the ILCOR 2005 guidelines (see Fig. 1). We assigned a numerical score to every resuscitation, following Carbine’s previously described method [16]: we awarded 2 points for every correct decision and proper procedure, 1 point for delayed interventions or inadequate technique, and 0 points for indicated procedures that were omitted or for inappropriate procedures (for details of how we scored each domain, see Table 1). The total score per resuscitation (“resuscitation score”) ranged from 4 to 22 points. A higher score indicated that more intense resuscitation was required. We obtained the percentage of adherence to the algorithm by dividing the score achieved (X) by the maximum possible score per patient that is, X of a potential of (4–22) points, as a percentage. We registered admission temperature and Apgar scores at 1, 5 and 10 minutes (min) as assigned by the caregiver and after video recording review.

**Table 1:** Data collection sheet

Patient’s identification:		Gestational age:	
Birth weight:		Sex:	
Twin? 1st or 2nd of 2		Time and date of birth	
C-section?			
Apgar score: assigned: 1 min: ᅟ5 min: ᅟ10 min: ᅟ			
Apgar score: camera: 1 min: ᅟ 5 min: ᅟ10 min: ᅟ			
Admission temperature: ᅟºC			
Analyzed aspects	0 points	1 point (any technical error in a correctly indicated maneuver is awarded 1 point; the main errors and examples are listed in every domain)	2 points
Heat loss prevention measures^a^	Not performed	No cap; baby dried with towels and then placed in a plastic wrap; if towels were used, they had to be replaced by new, preheated ones	Well done (dried and towels replaced OR plastic wrap)
Head in a “sniffing position”^a^	Not performed	Head in hyperextension or bent or to a side	Well done
Suctioning	Not performed when indicated	Done after the first 20 s; for more than 5 s; incorrect order (nasal suction before oral); incorrect suction catheter (not 8 F); excessively introduced catheter (more than 10 cm)	Well done
Stimulation	Not performed when indicated: inactive, apneic or not spontaneously breathing, or gasping, or bradycardic	Stimulation performed on other places than the back or the soles of the feet. Too aggressive (not gentle rubbing)	Well done
Preductal PO probe	Not placed in a baby who needed CPAP, PPV or oxygen	Not preductal (left hand or wrist, foot)	Preductal (right hand or wrist)
Administration of oxygen	Not used in a baby who needed it	Given free-flow oxygen; not administered with PPC or PPV; not discontinued when color or SpO_2_ improved; use of initial FiO_2_ other than 0.3	Well done
Administration of CPAP	Mandatory if < 28 weeks GA or ≥ 29 with a positive initial evaluation but distress	Evident mask leak; incorrect mask/cannula size	Well done
Administration of mask PPV	Not performed when needed	Initiation after the first 20 s; use of a self-inflating bag instead of an automatic or manual ventilator; incorrect mask size; incorrect rate (not 40-60 rpm); mask leak; not re-evaluated for response (HR and color) after 30 s)	Well done
Intubation	Not performed when needed	Duration of each intubation attempt (time from the introduction of the laryngoscope blade to the mouth to its removal) > 30 s); incorrect size of the endotracheal tube; position of the endotracheal tube not checked (auscultation/chest wall rise/inserted to correct depth); lack of ventilation between intubation attempts, Number of intubation attempts; Unplanned extubation	Well done
Chest compressions	Not performed when needed	Incorrect method (other than 2 thumbs or 2 fingers); incorrect area (other than lower third of the sternum); incorrect depth (not one third of the anterior-posterior diameter of the chest); incorrect rate (not 90 bpm); incorrect coordination with ventilation (not 3:1); initiation without correct ventilation; Not re-evaluated for response	Well done
Epinephrine administration	Not performed when needed	Not administered after 30 s of CC if heart rate < 60 bpm; Dose and route of administration	Well done
Surfactant administration	Not performed when indicated: intubated and < 28 or ≥ 29 weeks GA and FiO_2_ ≥ 0.3	Not administered at 10 min of life; Dose	Well done
Total points			

^a^Always mandatory

If PPV, CC or drugs are necessary, breathing, heart rate and color must be reassessed every 30 s.

*Min* Minutes, *PO* Pulse-Oximeter, *CPAP* Continuous Positive Airway Pressure, *PPV* Positive Pressure Ventilation, *GA* Gestational Age, *CC* Chest Compressions

We compared two groups of resuscitators: staff neonatologists (group N) and pediatricians on-call (group P). Neonatologists on-call performed a few of the resuscitations after-hours.

### Statistic analysis

We present the characteristics of our study population and its subgroups using the median, standard deviation (SD) and range for quantitative variables (gestational age, birth weight, temperature at admission and adherence to the algorithm and resuscitation score). We confirmed homogeneity of our subgroups in terms of gestational age, birth weight and resuscitation score. We used the Shapiro-Wilk test to evaluate normality in our subgroups. We compared normally distributed quantitative variables with a paired T-test between our two groups. For nonnormally distributed quantitative variables, we used the Mann-Whitney U test to compare the two groups.

We compared percentages of adherence to the algorithm for every domain with the Chi-square test for large groups and with Fisher’s exact test for smaller groups (fewer than 5 cases). Linear and logarithmic regressions were drawn to characterize the pattern of adherence to the algorithm.

We compared assigned Apgar scores with those given after analyzing the recordings and described them by their median and interquartile range (IQR). We measured interrater agreement between Apgar scores with Cohen’s kappa coefficient.

Statistical analysis was performed using SPSS (SPSS for Windows, V.20, Chicago, Illinois, USA). A *p*-value < 0.05 was considered significant.

## Results

Between April 2008 and August 2009, 162 infants < 32 weeks GA were born in our center. We analyzed 50 resuscitations (30.6%), a representative sample of the population. Groups N (staff neonatologists) and P (pediatricians on-call) were homogeneous. Tables 2 and 3 show the characteristics of our population and subgroups.

**Table 2:** Characteristics of our population and neonates < 32 weeks GA born during the study period

Characteristic	Study patients (*n* = 50)	Neonates < 32 weeks GA born during the study period (*n* = 162)	P^c^
Gestational age, SD (weeks) (range)	29^4^ ± 2^5^ (25^5^–31^6^)	29^1^ ± 2 (24^1^–31^6^)	NS (0.24)^a^
Male (%)	26/50 (52%)	88/162 (54.3%)	NS (0.07)^b^
BW, SD (g) (range)	1181 ± 368 (460–2015)	1201 ± 377 (340–2475)	NS (0.75)^a^
Twins (%)	19/50 (38)	81/162 (50)	NS (0.13)^b^
BW < 1500 g (%)	42/50 (84)	131/162 (80.8)	NS (0.61)^b^
C-section (%)	33/50 (66)	94/162 (58)	NS (0.31)^b^

*SD* Standard Deviation, *BW* Birth Weight, *GA* Gestational Age. ^a^Paired T- test, ^b^Chi-square, ^c^Indicates significance at the *P* < 0.05 level. NS: non-significant

**Table 3:** Subgroups in our study

Characteristic	Group N (staff neonatologists) (*n* = 18)	Group P (pediatricians oncall) (*n* = 32)	P^d^
GA (weeks, SD) (range)	29^4^ ± 1^6^ (25^5^–31^6^)	29^3^ ± 1^4^ (26^0^–31^6^)	NS (0.90)^a^
Male (%)	8/18 (44.4)	18/32 (56.2)	NS (0.61)^b^
BW (g, SD) (range)	1091 ± 418 (460–1900)	1232 ± 333 (720–2015)	NS (0.20)^a^
RS (possible points) (range)	12.66 ± 4.39 (6–20)	13.96 ± 3.71 (6–20)	NS (0.29)^c^

*Group N* Staff Neonatologists, *group P* Pediatricians On-call, *GA* Gestational Age, *SD* Standard Deviation, *BW* Birth Weight, *RS* Resuscitation Score. ^a^Paired T-test, ^b^Chi-square, ^c^Mann-Whitney U test, ^d^Indicates significance at the *P* < 0.05 level. NS: non-significant

Global adherence to the algorithm was 80.9 ± 14.2%, with no differences between groups N and P (81.5 ± 12.7% in group N versus 80.7 ± 15.0% in group P, *P* = 0.93, Mann-Whitney U), and was independent of the number of interventions required. Eight resuscitations (16%) were technically correct; 15/50 (30%) failed in one domain; 12/50 (24%) in two; 5/50 (10%) in three; 6/50 (12%) in four; 2/50 (4%) in five; and 2/50 (4%) in seven. The mean (SD) resuscitation score was 13.5 (3.9) points/resuscitation (range: 6–20). Table 4 analyzes the adherence to the algorithm by domains.

**Table 4:** Adherence to the algorithm

Domain	Indicated (%)	Performed (%)	Adherence to the algorithm (%) (PO/TTPx100)			
			Global	Group N	Group P	p^3^
Heat loss prevention	100 (50/50)	100 (50/50)	62 (31/50)	66.7 (12/18)	59.4 (19/32)	NS (0.84)^2^
Head in a “sniffing” position	100 (50/50)	94 (47/50)	94 (47/50)	94.4 (17/18)	93.7 (30/32)	NS (0.71)^1^
Clearing the airway	96 (48/50)	96 (48/50)	62.5 (30/48)	55.6 (10/18)	66.7 (20/30)	NS (0.59)^2^
Stimulation	64 (32/50)	30 (15/50)	93.3^4^ (14/15)	80 (4/5)	100 (10/10)	NS (0.33)^1^
Placing a preductal pulse-oximeter probe	82 (41/50)	90.2 (37/41)	63.4 (26/41)	76.9 (10/13)	57.1 (16/28)	NS (1.49)^1^
Administration of oxygen	68 (34/50)	68 (34/50)	94.1 (32/34)	100 (10/10)	91.3 (21/23)	NS (0.48)^1^
CPAP	60	60	100	100	100	–
Administration of PPV	60 (30/50)	96.7 (29/30)	79.3 (23/29)	60 (6/10)	85 (17/20)	NS (0.14)^1^
Intubation	32 (16/50)	87.5 (14/16)	0 (0/16)	0 (0/5)	0 (0/11)	–
CC	4 (2/50)	2 (1/50)	0	0	0	–
Epinephrine administration	?	4 (2/50)	0	0	0	–
Surfactant administration	24 (12/50)	20 (10/50)	100^4^ (10/10) 83.3 (10/12)^5^	75 (3/4)	87.5 (7/8)	NS (0.58)^1^

*PO* Points Obtained, *TPP* Total Possible Points, *Group N* Staff Neonatologists, *Group P* Pediatricians On-call, *CPAP* Continuous Positive Airway Pressure, *PPV* Positive Pressure Ventilation, *CC* Chest Compressions. ^1^Fisher’s exact test, ^2^Chi-square, ^3^Indicates significance at the *P* < 0.05 level, ^4^when done, ^5^when indicated. NS: non-significant

Table 5 shows results from measures to prevent heat loss and its relation to admission temperature. We found no differences between the group that received correct measures to prevent hypothermia and the group that did not. Intubation differentiated intensive (16–20 total possible points) from mild resuscitation (6–16 points). Infants who did not need intubation (*n* = 36) had a mean global adherence to the algorithm of 83%. Deviations from the algorithm in this group did not correlate with the intensity of resuscitation (R^2^ = 0.0013).

**Table 5:** Heat loss prevention

Group	Heat loss prevention adherence to algorithm (%) (PO/TPP)	P^c^	Admission temperature (C) (range)	P^c^
Total	62 (31/50)		36.0 ± 0.6 (34.6–37.8)	
Group N	66.7 (12/18)	NS (0.84)^a^	35.8 ± 0.7 (34.6–37.6)	NS (0.09)^b^
Group P	59.4 (19/32)		36.1 ± 0.6 (35.0–37.8)	
Correct heat loss prevention measures (*n* = 31)			36.1 ± 0.7 (35.0–37.8)	NS (0.60)^b^
Incorrect heat loss prevention measures (*n* = 19)			36.0 ± 0.6 (34.6–36.9)	

^a^Chi-square

^b^Paired T-test

^c^Indicates significance at the *P* < 0.05 level. *NS* non-significant, *PO* Points Obtained, *TPP* Total Possible Points, *Group N* Staff Neonatologists, *Group P* Pediatricians On-call

### Some errors we observed

#### Heat loss prevention

Twelve percent of patients were placed in plastic wrap after drying. When only dried, the technique was correct in 58.1% (18/31) of cases; 22.6% (7/31) did not have the towels changed, and 19.3% (6/31) had no cap. Only 22.4% of patients (11/49) were normothermic (36.5–37.5 °C); 73.5% (36/49) were hypothermic. More critically ill patients were more likely to receive worse anti-hypothermia measures because they were being subjected to other procedures: ventilation, intubation, chest compressions (CC) and surfactant administration. Sixty-eight percent of patients (13/19) in whom heat loss prevention was incorrect had a resuscitation score ≥ 14 points, which means that they received at least ventilatory support.

#### Clearing the airway with a suction catheter

The following errors were observed: oral without nasal suctioning, 16.7%; undue suction (over 5 s, range 31–50 s), 8.3%; use of a larger catheter than recommended, 8.3%; delayed suctioning after 20 s, 6.2%, or after ventilation, 4.2%; incorrect suctioning order (first nasal), 2.1%; and excessive introduction of the catheter, 2.1%. We observed no episodes of severe bradycardia during suctioning.

#### Stimulating breathing

One baby was stimulated when unnecessary, and 32% (16/50) who needed stimulation did not receive it, particularly those in worse condition. One baby had his face rubbed.

#### Administration of PPV

One patient was intubated without previous PPV. We observed the following errors: undue delay in starting PPV (at 56, 60 and 69 s) in 10.3% of cases (3/29), use of a self-inflating bag instead of a ventilator in 6.9% of patients (2/29), ventilation without previous suctioning when airway was obstructed in 6.9% (2/29), lack of ventilation between intubation attempts in 3.4% (1/29), and face mask leak in 3.4% (1/29). We did not use a respiratory function monitor, so we could not objectively document leaks; however, in one patient, the lack of a mask seal was obvious. In some patients, more than one mistake occurred.

#### Intubation

All intubations deviated from the algorithm. Two of 16 (12.5%) patients could not be intubated after several attempts; their indication for intubation was respiratory distress. They were transferred to the Neonatal Intensive Care Unit (NICU) with CPAP and intubated under sedation. The mean number of intubation attempts was 2.2 (range 1–6); success on the first attempt happened in 33.3% of cases; on second attempt, 38.9%; on third attempt, 16.7%; and 11.1% needed more than three attempts (5 and 6). We analyzed 40 intubation attempts. Two unplanned extubations after surfactant administration (due to incorrect securing of the tube) required reintubation. In all intubation cases, at least one attempt took longer than recommended (30 s). Mean duration to perform intubation was 58.8 ± 23.4 s (range 17–128 s). Only 5 of 40 intubations (12.5%) were achieved within 30 s.

#### Chest compressions and epinephrine administration

CC technique was correct, but it was initiated late. Despite correct intubation and ventilation, one newborn was bradycardic at 3:59 min, and CCs were started at 7:22 min. One patient received epinephrine without previous CCs, and another received epinephrine when CCs were started.

The median (IQR) assigned Apgar scores at 1, 5 and 10 min were 7 (5.7–9), 9 (8–10) and 10 (8–10). The median (IQR) Apgar scores after reviewing NRs were 7 (5–9), 9 (6.7–10) and 9 (7.7–10). Agreement at the three time points was acceptable (Kappa coefficient 0.35). Interrater reliability in evaluating Apgar scores was moderate at 1, 5 and 10 min (Cohen’s kappa coefficients: 0.57, 0.60 and 0.44, respectively).

**Fig. 2:**
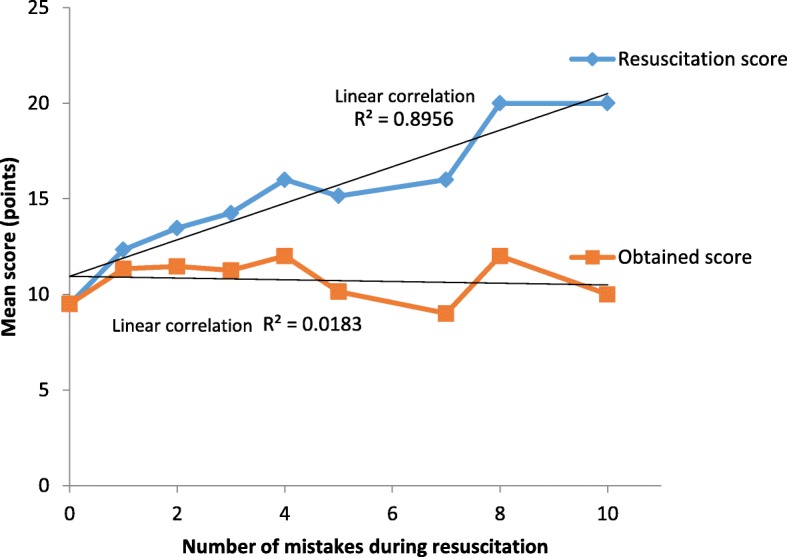
Correlation between the number of errors during resuscitation and the mean obtained resuscitation score (red line) and the maximum resuscitation score (blue line). The difference between the blue and red lines was the average of virtually lost points

**Fig. 3:**
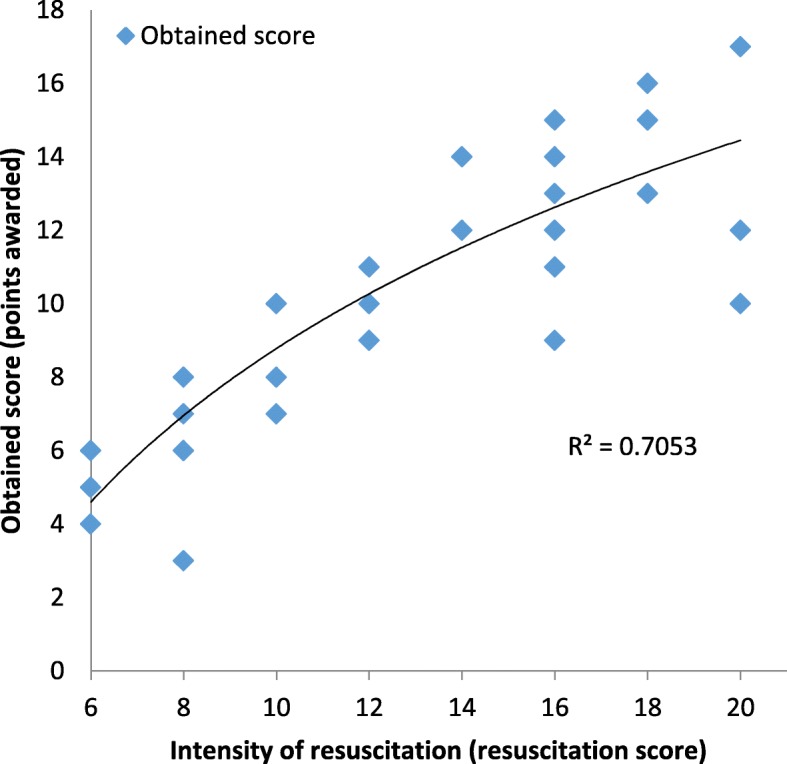
Relationship between intensity of resuscitation and obtained score

Our resuscitation team obtained a median of 10 points per resuscitation (red line), regardless of the resuscitation score (blue line), which means that resuscitations with a resuscitation score above 10 were poorly done (Fig. 2). Figure 3 shows that the relationship between the resuscitation score and the points obtained was nearly logarithmic (R^2^ = 0.7053), which means that resuscitators scored very few additional points as the resuscitation intensity increased.

## Discussion

All our patients were resuscitated in our dedicated room for resuscitation, which provides a setting similar to that in the NICU. Vento [4, 6] suggested that incorporating an intensive care environment into the DR could enhance survival and reduce the morbidity of extremely low birth weight (BW) infants. Among our study population, we found a high percentage of hypothermia (73.5%), which led us to make some changes in our resuscitation room to reduce hypothermia: we increased the temperature to 26 °C by keeping the doors locked and installed a heater next to the resuscitation cot. We use heated, humidified gases for ventilation.

Several authors have proven that performance often deviates from guidelines. Our study is the first report on adherence to the neonatal resuscitation algorithm for very preterm infants in a tertiary care center in Spain.

Carbine was the first to use video recording to evaluate NR [16]. We based our study on his publication and adopted his scoring system. Carbine found deviations in 54% of NRs. We evaluated more aspects and may have detected more errors (84%). We believe that our resuscitation score was higher: 22% of Carbine’s patients only required stimulation (whereas 64% of ours needed stimulation); 80% required stimulation and oxygen, and only 7% needed PPV (vs our 80% respiratory support and 32% intubation). Carbine reported errors in the mask ventilation rate. We considered the use of a self-inflating bag an error, as we used an automatic ventilator. Consequently, we did not find this error. Only 28.6% of Carbine’s cases involving PPV had no deviations, which is worse than our 72.7% rate of proper ventilation. Among Carbine’s infants, 58.3% were intubated on the first attempt (vs our 33.3%), and only 33.3% (vs our 87.5%) were intubated within the established time limit. Like Carbine, we observed that perfect resuscitations were more likely for less intense interventions. None of our patients who required intubation received a perfect resuscitation.

Similar to Dekker [17], we observed that stimulation was often indicated but not performed, and when it was applied, it was most often indicated. Dekker’s infants who received no stimulation required intubation more often (18 vs 7%); in our case, 62.5% of intubated infants had not been stimulated and 25% of stimulated infants did not require intubation. However, 18.7% of infants (6/32) who were stimulated were also intubated afterward.

By using video recording at a Nepalese tertiary hospital, Lindbäck [10] identified deviation from guidelines in over 50% of resuscitations. Most errors concerned the use of bag-and-mask ventilation (which we did not evaluate, as ventilating with a bag and mask was an error in our study), suction and excessive use of oxygen. Their results seem more favorable than ours. However, Lindbäck did not focus on preterm infants.

Gelbart [8] reported that the demanding technical skills scored higher than the more basic steps of resuscitation because technique is taught, whereas clinical assessment, communication skills and teamwork need practice. He found that invasive ventilation and surfactant administration were best performed, with median scores of 100%, whereas the performance of preparation and initial steps (69%) and assessment and communication of heart rate (75%) was worse. In our patients, most errors took place during the initial steps as well, whereas administration of CPAP, PPV and surfactant were performed better. Surfactant was administered in 83.3% of our cases when indicated. Technique was always correct, although two patients who required it did not receive it; the PO was not functioning, and the pediatrician preferred to administer it in the NICU with proper monitoring.

Schilleman [9] used video recording to evaluate compliance with NR guidelines in a population similar to ours, although our patients were in better conditions according to the Apgar scores. Schilleman found that deviations mainly occurred within the first 30 s because caregivers needed more time to perform the initial steps and mainly involved the way ventilation was given. As such, Schilleman suggested that 1 min be allowed for the initial evaluation, which is what the current ILCOR guidelines allow.

No intubations were perfectly performed. We analyzed 40 intubation attempts. Success occurred on the first attempt in 33.3% of cases and on the second attempt in 38.9%; more than three attempts (5 and 6) were required in only 11.1% of cases. In all intubation cases, at least one attempt took longer than recommended. Only 12.5% of intubations took place within the allotted 30 s. Other authors have reported similar deficiencies. Lane [18] reported a mean duration for successful attempts of 27.3 s; 30% infants were intubated on the first attempt, 30% on the second, 20% on the third, and 20% required more than three. Success was higher for 30 s, and no infants decompensated between 20 and 30 s; 20% of successful attempts took longer than 40 s. Finer and Rich’s [3, 15] overall success rate for intubation was 33% within the allotted 20 s and 56% within 30 s. They reported an average of at least three attempts to successfully intubate infants < 1000 g. Our intubation success rate was higher than that reported by Finer and Rich. We needed, on average, 2.2 attempts to intubate infants < 1000 g (range 1–5), but unfortunately, it took longer (median (SD) 58.1 s (23.4), range 17–128 s). O’Donnell [19] analyzed intubation attempts in 31 infants (mean GA 28 weeks and BW 1227 g). Intubation attempts were often unsuccessful and successful attempts often took more than 30 s (17% were successful within 20 s, 20% between 20 and 29 s, and 25% > 30 s). Konstantelos [7] needed a median of 2 attempts, and 47 (25–60) s for intubation, and only 11% were successful within the allotted time. Wozniak [20] analyzed intubation attempts in preterm infants (795 g median BW, 25 weeks’ GA) and reported a mean duration of 35 s and 2 attempts. Like Konstantelos [21], we believe that the lack of medication for intubation and surfactant administration are the reasons for the longer time needed for intubation. Because health care providers often underestimate the passage of time during NR, it is difficult to realize when the allotted time has passed. The American Academy of Pediatrics NRP used to allow 20 s for intubation, but since several studies reported that it often took longer [3, 16, 18, 19, 21] and that infants did not decompensate between 20 and 30 s, the current limit is 30 s [20].

As Fig. 2 shows, resuscitators obtained, on average, 10 points per patient regardless of the intensity of resuscitation, which means that caregivers did not score more points in more complex resuscitations. There are two reasons for the constant red line: a) some initial, common mistakes in heat loss prevention, suctioning and a postductal PO probe placement prevented most mild NRs (mean resuscitation score of 13 points) from scoring higher, and b) the points corresponding to almost all complex domains (intubation, chest compressions or epinephrine administration) were lost. The consequence is the flat line in the relationship between mistakes and the obtained score. However, the more mistakes that occur in a NR, the higher the resuscitation score (blue line, R^2^ = 0.895), meaning that complex domains (the area above the red line) are lost in terms of obtained points. Not surprisingly, the relationship between the resuscitation score and the obtained score (Fig. 3) followed a strong logarithmic pattern (R^2^ = 0.705). This finding means that although more points are possible, the resuscitator team would gain little benefit from those complex domains. More emphasis must be placed on the initial steps, which are common to most NRs, and especially on training for complex skills such as intubation.

This study has some limitations. Its purpose was to evaluate adherence to NR guidelines. For this reason, all mistakes counted equally, although it is obvious that not all the deviations are equally serious: some are mild and nontranscendental (for example, duration of oral suctioning) whereas others are potentially harmful (like timing and route of epinephrine administration). The same mistake could have consequences or not, depending on the patient. For example, placing a postductal PO probe in a patient who does not receive oxygen or ventilatory support has no impact on the maneuvers performed but may lead to hyperoxia in an intubated neonate. Only 16% of our resuscitations perfectly followed the algorithm, but the number of mistakes per resuscitation was low, and global adherence to the algorithm was 80.9%. We acknowledge that the value of global adherence in itself has little meaning without the proper analysis of the main and more critical errors. Another limitation of our study is the lack of feedback of our findings to the resuscitation team. We designed the study to assess adherence to the algorithm and find the most common errors. In the pilot study, we did not consider an active intervention with the resuscitation team. Sharing our findings with them would probably improve performance. In most cases, the resuscitation team consisted of two neonatologists or two pediatricians on-call. All of them are trained in neonatal resuscitation, although their expertise varies from more than 30 years to only a few months after completing a residency. However, 38% of our patients were twins, which may worsen performance, as in some cases, there was only one caregiver per patient [22]. All our medical staff was aware of this study when it started, and we periodically reminded them about it. All the neonatologists and pediatricians who work at our hospital participated in this study. The medical staff turned the video camera on, automatically consenting to be recorded, when they were called to the DR. Recording usually began minutes before the neonate was born but sometimes began when the newborn arrived at the resuscitation room.

Similar to many other previous studies, our study demonstrated that deviations from the algorithm exist. Many of the errors have to do with timing: some maneuvers take longer than allotted, and personnel are not aware of this [23].

We compared performance of staff neonatologists with pediatricians on-call. We thought that the neonatologists would perform better since they work with only newborns and are subspecialized in neonatology, whereas after-hour on-call pediatricians who cover this shift often do not work with only newborns. We observed no differences between these two groups, which means that we have a good team of pediatricians who perform as well as neonatologists. This is a positive aspect to consider. Although global adherence to the algorithm was high, mistakes were common despite our staff’s training.

In line with other authors [8, 22–25], we found a discrepancy in Apgar scores, particularly when the Apgar was not 9/10/10, and the staff attending the delivery commonly overestimated the score. It is easy to score 9/10/10 if no resuscitation is necessary, whereas it is difficult to remember the patient’s situation at 5 and 10 min when resuscitation is required. Video recording scores tended to be lower than scores given by neonatologists (47.1% at 1 min, 73.3% at 5, 88.9% at 10). Gelbart [8] also found overestimation of Apgar scores by a median value of 2 points at 1 and 5 min. As other authors suggest, we believe that memories of a stressful past event can be inaccurate, and Apgar scores are usually calculated afterward [24].

Finally, we are aware that our study sample was small. We aimed to analyze 50 resuscitations due to the difficulty in obtaining written consent before resuscitation started. Nonetheless, we managed to record one-third of our potential cases, which is a significant sample. The ILCOR guidelines have changed twice since we conducted this study, and some actions that we considered mistakes would now be correct, for example, supporting transition rather than keeping timing strict or not suctioning routinely. While it is true that our data are old, and a few aspects are outdated, our aim was to assess our performance in terms of adherence to the algorithm, that is, if our physicians performed according to the written rules, not the appropriateness of the algorithm. Our results would probably be similar today. Even though only one person reviewed the recordings, the camera was in a good position, and the scoring system was clear, so this bias is likely minimal. There was no feedback given to the resuscitation team during the study period, but our findings could serve as both a starting point for further studies and a teaching tool. As far as we know, there are no similar studies published in Spain to date.

## Conclusions

Resuscitation of very preterm newborns often deviates from guidelines. Perfectly performed resuscitations are infrequent, although global adherence to the algorithm is high. Resuscitations led by pediatricians on-call and neonatologists are performed equally correct. Intubation training may improve complex resuscitations the most.

## Abbreviations

bpm: Beats Per Minute
BW: Birth Weight
C: Celsius
CC: Chest Compressions
CPAP: Continuous Positive Airway Pressure
DR: Delivery Room
GA: Gestational Age
ILCOR: International Liaison Committee on Resuscitation
min: Minute
NICU: Neonatal Intensive Care Unit
NR: Neonatal Resuscitation
NRP: Neonatal Resuscitation Program
PO: Pulse-Oximeter
PPV: Positive Pressure Ventilation
s: Second
SD: Standard Deviation
Temp: Temperature
